# Supplementary material for: A multi-scale clutch model for adhesion complex mechanics
Source: PLoS Comput Biol. 2023 Jul 14;19(7):e1011250. doi: 10.1371/journal.pcbi.1011250 (PMC10393167; doi:10.1371/journal.pcbi.1011250)
Supplement: S1 Text — Number of myosin motors and stall force. Extended data availability. Fig A1. Effect of slip and catch bonds in cell adhesion behavior. Fig A2. Effect of reinforcement by talin unfolding in cell adhesion. Fig A3. Timeevolutionoftheslipcase. Fig A4. Timeevolutionofthecatchcase. Fig A5. Number of equispaced ligands nc as a function of the adhesion radius. Fig A6. Comparison of the number of MC simulations for two different final times tf = 100s and tf = 1000s. Fig A7. Mean and standard deviation of the force Fc (pN) computed over bound binders. Fig A8. Sensitivity analysis of the reinforced case. Table A1. Model parameters for slip and catch cases. Table A2. Parameters for myosin motors used inside Eqs. 1, 2 and 3, with experimental references. (PDF) [file pcbi.1011250.s001.pdf]

Supporting Information:
A multi-scale clutch model for adhesion complex mechanics

Chiara Venturini [1], Pablo Sáez[1,2,3*]

1 Laboratori de Càlcul Numèric (LaCaN), Universitat Politècnica de Catalunya, Barcelona, Spain.
2 E.T.S. de Ingeniería de Caminos, Universitat Politècnica de Catalunya, Barcelona, Spain
3 Institut de Matemàtiques de la UPC-BarcelonaTech (IMTech), Universitat Politècnica de Catalunya, Barcelona, Spain

*pablo.saez@upc.edu

List of Figures

A1 **Effect of slip and catch bonds in cell adhesion behavior.** 7
A2 **Effect of reinforcement by talin unfolding in cell adhesion.** . . 8
A3 **Time evolution of the slip case.** 9
A4 **Time evolution of the catch case.** 10
A5 **Number of equispaced ligands n_c as a function of the adhesion radius.** 11
A6 **Comparison of the number of MC simulations for two different final times $t_f = 100\,\mathrm{s}$ and $t_f = 1000\,\mathrm{s}$.** 12
A7 **Mean and standard deviation of the force F_c (pN) computed over bound binders.** 12
A8 **Sensitivity analysis of the reinforced case.** 13

List of Tables

A1 Model parameters for slip and catch cases. All model parameters are taken from previous works [1, 2]. 2
A2 Parameters for myosin motors used inside Eqs. 1, 2 and 3, with experimental references. 3

Results of the clutch model

We first analyze cell adhesion in terms of previous clutch models. We consider ACs crowded with integrins expressing either slip [1] or catch [2] bonds, which we refer to as slip and catch cases from now on. In what follows, and unless specified otherwise, we take model parameters for the on/off rates of slip and catch bonds from literature (see Methods, Table A1). We take values of a slip bond (see Eq. 4) that reproduced experimental data of chick forebrain neurons [1]. To reproduce cells crowded with catch bonds (see Eq. 5), we use model parameters that reproduced the lifetime of $\alpha_5\beta_1$ integrins at its maximum activation state in the presence of Mn^{2+} ions [3]. For all

simulations in this section, we fix the radius of the AC to a =1700 nm. The number of ligands is n_c =1200 in the catch case [2], which results in a ligands spacing of d = 100 nm (see Fig. A5 for details). To seemingly compare slip and catch cases, we keep d = 100 nm constant for the slip case, and fixing n_c =75 [1] we obtain an AC radius of a = 447 nm. The remaining model parameters are summarized in Table A1. We analyze the model results in terms of the substrate stiffness, with Young's modulus in the range of 0.1 - 100 kPa. This is the stiffness range found in biological tissues [5] and on which most in vitro studies have focused (see, e.g., [1, 2]). We analyze the probability of bound binders P_b, the actin velocity v, the cell traction P, the maximum and the average force over the bound binders, F_c^{max} and $F_{c,bound}$, the maximum and the average displacement for bound binders x_{int}^{max} and $x_{int,bound}$, and the displacement of the substrate x_{sub}. We average in time all these variables of interest to obtain the model predictions at specific stiffnesses of the substrate.

	E	a	$F_{b,slip}$	F_m	k_c	$k_{off,slip}$	k_{ont}	d_{int}	n_c	v_u	n_m
	kPa	nm	pN	pN	pN/nm	s^{-1}	μ m^{2}s	intμ m^2		nm/s	
**Slip**[1]	0.1 - 100	447	2	2	5	0.1	1	1	75	120	75
**Catch** [2]	0.1 - 100	1700	-	2	1000	-	0.0002	300	1200	110	800

Table A1. Model parameters for slip and catch cases. All model parameters are taken from previous works [1, 2].

The results for the slip and catch cases are in Fig. A1. Our simulations reproduce previous results [1, 2, 4]. To better understand the model behavior, we analyze the time evolution of all model variables (see Figs. A3 and A4 for details). We specifically focus on the lifetime of each adhesion cycle, that is the average time from the formation of the AC until its complete disengagement. The lifetime of the AC of both slip and catch cases decreases as the stiffness of the substrate increases. In the slip case, the average length of the cycle for E = 1 kPa is 7.27 s and for E = 10 kPa is 0.215 s. In the catch case, for E = 1 kPa it is 4.25 s and for E = 10 kPa it is 0.344 s.

We also review recent developments that have extended the original clutch model introducing the effect of talin and vinculin reinforcement [2, 6] (see Fig. A2 for details). Talin unfolding is a mechanosensing event triggered by force that exposes one VBS, where vinculin binds at a force-independent rate k_{onv}. The unfolding rate of talin, k_{unf}^*, responds to force according to Bell's model as a slip bond. Talin refolding also depends on force [2]. If talin unfolds, it can either refold again or vinculin can bind to it, increasing integrin density by $int_{add} = 24$ integrins/μm^2. The binding rate of integrins follows $k_{on} = k_{ont} d_{int}$, where k_{ont} is the true binding rate characterizing each integrin-fibronectin bond, and d_{int} is the density of integrins in the AC. If the clutch unbinds before vinculin binds to talin, integrin density is decreased by int_{add}, reflecting the fact that adhesions lose integrins if force application is decreased [7, 8]. In the model, integrin density is never allowed to go below the initial value nor above a maximum integrin density, $m_r = 15x10^3$ integrins/μm^2, as there is a limiting density of integrins, or separation between integrins, in the AC (see Fig. A5 for further details). Again, our results reproduce previous experimental and theoretical results [6, 2].

Number of myosin motors and stall force

To find values for the number of myosin motors n_m attached to an actin fiber and the stall force for a single myosin motor F_m, we look for an experimental value of the total stall force $F_{stall} = n_m\, F_m$. The ensemble stall force can be written as [9]:

$$F_{stall} = F_{sm}\, N_{heads}\, \rho(F_{sm}), \tag{1}$$

where $F_{sm} = K_{x-bridge} d_{step}$ is the stall force for a single motor, N_{heads} is the number of myosin motors, and $\rho(F_{sm})$ is the duty ratio of a single motor at the stall, which can be computed as:

$$\rho(F_{sm}) = \frac{k_{on}}{k_{on} + k_{off}(F_{sm})}. \tag{2}$$

The expression for the myosin off-rate is [10]:

$$k_{off}(F) = k_{off}(0) \left[\alpha_{catch} \exp\left(\frac{-F x_{catch}}{k_B T} \right) + \alpha_{slip} \exp\left(\frac{F x_{slip}}{k_B T} \right) \right]. \tag{3}$$

Myosins are a superfamily of motor proteins. Myosin II is responsible for producing muscle contraction in muscle cells in most animals, but it is also found in non-muscle cells inside stress fibers. Hence, it can be further classified into skeletal muscle myosin II, smooth muscle myosin II, and nonmuscle myosin II (NM II) [9]. More precisely, the myosins having a role in cell adhesion are NM IIA and NM IIB. NM IIA mediates the initial maturation of FAs, and NM IIB is found in fibrillar adhesions. Adhesions with NM IIA are dynamic, while those with NM IIB are very stable [11]. In the leading edge of the cell, both types are present, while NM IIA characterizes the rear edge, and NM IIB is found in the actin close to the nucleus of the cell [12]. It has been demonstrated that 90% of the traction force generated by mouse embryonic fibroblasts (MEFs) on a fibronectin-coated substrate is lost with the removal of NM IIs and that NM IIA is responsible for $\approx 60\%$ of the force, whereas NM IIB accounts for the $\approx 30\%$ [13, 14].

**Parameters**		
$K_{x-bridge}$(pN/nm)	0.7	[15]
d_{step} (nm)	5.5	[15]
N_{heads}	50	[16]
k_{on} (s^{-1})	0.2	[17], [18]
$k_{off}(0)$ NM IIA (s^{-1})	1.71	[17]
$k_{off}(0)$ NM IIB (s^{-1})	0.35	[18]
α_{catch}	0.92	[10]
α_{slip}	0.08	[10]
x_{catch} (nm)	2.5	[10]
x_{slip} (nm)	0.4	[10]

Table A2. Parameters for myosin motors used inside Eqs. 1, 2 and 3, with experimental references.

Using Eqs. 1, 2 and 3, with parameters in Table A2, we compute the ensemble stall force F_{stall} for NM IIA and NM IIB. Considering that in our setting there is a percentage of myosin motors of each type, we choose a total stall force in the range $[F_{stall}^{NMIIA}, F_{stall}^{NMIIB}] = [73.34, 143.03]$ pN, and precisely $F_{stall} = 80\,\text{pN}$. The stall force for a single myosin motor is found to be at least $F_m = 1.7\,\text{pN}$ [20], therefore, following $F_{stall} = n_m\, F_m$, we consider $n_m = 40$ myosin motors, each with a stall force $F_m = 2\,\text{pN}$.

Supplementary figures

Extended figures are uploaded in https://doi.org/10.5281/zenodo.7907673. Specifically:
- All the figures of the model results in time for all substrates rigidities in Sections "Role of the ECM rigidity in $\alpha_5\beta_1$ and $\alpha_V\beta_3$-crowded focal adhesions" and "Adhesion dynamics in $\alpha_5\beta_1$ and $\alpha_V\beta_3$-based adhesion complexes".
- All the figures of the model result in Section "Integrins behavior in AC dynamics".

- All the figures of the model result in Section "Variations in AC behavior due to ligand spacing".

The code used in this section to model $\alpha_5\beta_3$ is also uploaded in https://doi.org/10.5281/zenodo.7906839.

References

1. C. E. Chan, D. J. Odde, Traction dynamics of filopodia on compliant substrates, Science 322 (5908) (2008) 1687–1691.

2. A. Elosegui-Artola, R. Oria, Y. Chen, A. Kosmalska, C. Pérez-gonzález, N. Castro, C. Zhu, X. Trepat, P. Roca-Cusachs, Mechanical regulation of a molecular clutch defines force transmission and transduction in response to matrix rigidity, Nature Cell Biology 18 (October 2015) (2016) 540.

3. F. Kong, A. J. García, A. P. Mould, M. J. Humphries, C. Zhu, Demonstration of catch bonds between integrin and its ligand, Journal of Cell Biology 185 (7) (2009) 1275–1284.

4. A. Elosegui-Artola, X. Trepat, P. Roca-Cusachs, Control of mechanotransduction by molecular clutch dynamics, Trends in Cell Biology 28 (5) (2018) 356–367.

5. C. F. Guimarães, L. Gasperini, A. P. Marques, R. L. Reis, The stiffness of living tissues and its implications for tissue engineering, Nature Reviews Materials 5 (5) (2020) 351–370.

6. A. Elosegui-Artola, E. Bazellières, M. Allen, I. Andreu, R. Oria, R. Sunyer, J. Gomm, J. Marshall, L. J. Jones, X. Trepat, P. Roca-Cusachs, Rigidity sensing and adaptation through regulation of integrin types, Nature Materials 13 (2014) 631.

7. N. Q. Balaban, U. S. Schwarz, D. Riveline, P. Goichberg, G. Tzur, I. Sabanay, D. Mahalu, S. Safran, A. Bershadsky, L. Addadi, Others, B. Geiger, Force and focal adhesion assembly: a close relationship studied using elastic micropatterned substrates., Nature cell biology 3 (5) (2001) 466–472.

8. D. Riveline, E. Zamir, N. Q. Balaban, U. S. Schwarz, T. Ishizaki, S. Narumiya, Z. Kam, B. Geiger, A. D. Bershadsky, Focal Contacts as Mechanosensors: Externally Applied Local Mechanical Force Induces Growth of Focal Contacts by an Mdia1-Dependent and Rock-Independent Mechanism, Journal of Cell Biology 153 (6) (2001) 1175–1186.

9. S. Stam, J. Alberts, M. L. Gardel, E. Munro, Isoforms confer characteristic force generation and mechanosensation by myosin II filaments, Biophysical Journal 108 (8) (2015) 1997–2006.

10. B. Guo, W. Guilford, Mechanics of actomyosin bonds in different nucleotide states are tuned to muscle contraction, Proceedings of the National Academy of Sciences of the United States of America 103 (2006) 9844–9849.

11. M. Vicente-Manzanares, A. R. Horwitz, Adhesion dynamics at a glance, Journal of Cell Science 124 (23) (2011) 3923–3927.

12. M. Vicente-Manzanares, X. Ma, R. S. Adelstein, A. R. Horwitz, Non-muscle myosin II takes centre stage in cell adhesion and migration, Nature Reviews. Molecular Cell Biology 10 (11) (2009) 778–790.

13. Y. Cai, N. Biais, G. Giannone, M. Tanase, G. Jiang, J. M. Hofman, C. H. Wiggins, P. Silberzan, A. Buguin, B. Ladoux, M. P. Sheetz, Nonmuscle myosin IIA-dependent force inhibits cell spreading and drives F-actin flow., Biophysical Journal 91 10 (10) (2006) 3907–3920.

14. M. S. Shutova, T. M. Svitkina, Common and Specific Functions of Nonmuscle Myosin II Paralogs in Cells, Biochemistry (Moscow) 83 (12) (2018) 1459–1468.

15. C. Veigel, J. Molloy, S. Schmitz, J. Kendrick-Jones, Load-dependent kinetics of force production by smooth muscle myosin measured with optical tweezers, Nature Cell Biology 5 (2003) 980–986.

16. R. Niederman, T. D. Pollard, Human platelet myosin II. In vitro assembly and structure of myosin filaments, The Journal of Cell Biology 67 (1975) 72–92.

17. M. Kovacs, F. Wang, A. Hu, Y. Zhang, J. R. Sellers, Functional divergence of human cytoplasmic myosin II: kinetic characterization of the non-muscle IIA isoform, Journal of Biological Chemistry 278 (40) (2003) 38132–38140.

18. F. Wang, M. Kovacs, A. Hu, J. Limouze, E. V. Harvey, J. R. Sellers, Kinetic mechanism of non-muscle myosin IIB: functional adaptations for tension generation and maintenance, Journal of Biological Chemistry 278 (30) (2003) 27439–27448.

19. P. Kanchanawong, G. Shtengel, A. M. Pasapera, E. B. Ramko, M. W. Davidson, H. F. Hess, C. M. Waterman, Nanoscale architecture of integrin-based cell adhesions, Nature 468 (7323) (2010) 580–584.

20. J. E. Molloy, J. E. Burns, J. Kendrick-Jones, R. T. Tregear, D. C. S. White, Movement and force produced by a single myosin head, Nature 378 (6553) (1995) 209–212.

21. R. Oria, T. Wiegand, J. Escribano, A. Elosegui-artola, J. J. Uriarte, C. Moreno-pulido, I. Platzman, P. Delcanale, L. Albertazzi, D. Navajas, X. Trepat, J. M. García-aznar, E. A. Cavalcanti-Adam, P. Roca-cusachs, Force loading explains spatial sensing of ligands by cells, Nature 552 (7684) (2017) 219–224.

22. R. Changede, X. Xu, F. Margadant, M. P. Sheetz, Nascent integrin adhesions form on all matrix rigidities after integrin activation, Developmental Cell 35 (5) (2015) 614–621.

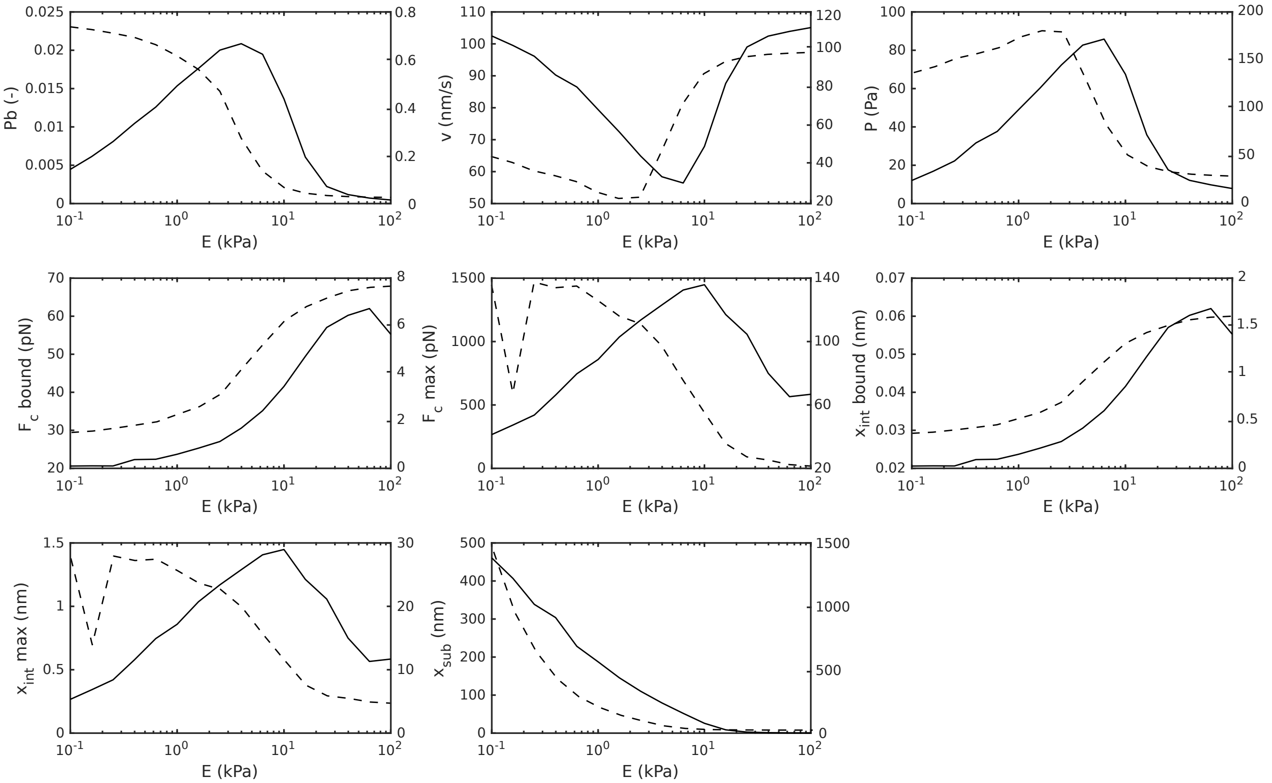

Fig A1. Effect of slip and catch bonds in cell adhesion behavior.
Model behavior for slip (dashed line, with corresponding y-axis on the right) and catch (solid line, with corresponding y-axis on the left) cases. Plot against Young's modulus of the substrate E of the variables P_b, v, P, F_c over bound binders, F_c^{max}, x_{int} over bound binders, x_{int}^{max} and x_{sub}. In the slip case, the probability of bound binders, P_b, decreases as the substrate stiffness increases. However, P_b shows a biphasic relation in the catch case, where it first increases and then decreases with increasing stiffness of the substrate. In absolute values, P_b almost doubles in the catch case with respect to the slip case. At large stiffnesses, the frictional slippage explains the reduction in the number of bound binders. The different P_b at low stiffnesses, where the load-and-fail cycle emerges, is due to the differences in the slip/catch bonds behavior. The maximum lifetime of slip bonds is at low forces (≈0-5 pN), while it maximizes at intermediate forces (≈30-35 pN) for catch bonds. Therefore, if the force at each clutch is low at low substrate rigidities, the model predicts larger binding/unbinding rates for the slip bonds than for catch bonds, which explains the difference in the number of bound binders at low stiffnesses. The same arguments explain the behavior of the maximum force F_c^{max} and maximum displacement x_{int}^{max} on the molecular clutches. The deformation of the substrate, x_{sub}, decreases as the stiffness of the substrate increase and vanishes at high stiffnesses because the increase in the mean force is not enough to compensate for the increase in substrate rigidity. The mean force, F_c^{bound}, and the mean displacement, x_{int}^{bound}, increase as the substrate stiffness increases. However, the maximum and average forces and displacements are one order of magnitude higher for the slip case than the catch case. The hill shapes in P_b and F_{sub}, as well as their magnitudes, explain the shape of the tractions, where the catch case shows a quick increase in cell traction at low rigidities and lower maximum values with respect to the slip case. When the cell traction is analyzed against the substrate stiffness, there is an optimal rigidity value that indicates the stiffness at which the cell traction is maximum. The differences in the optimal stiffnesses are controlled by the lifetime of the slip and catch bond dynamics. The retrograde flow v follows the same tendency but is opposite to the cell tractions. The velocity is maximum when the cell traction is zero (no adhesion) and, therefore, there is no resistance to the flow, while the velocity is minimum when the tractions are maximum (i.e. at the optimal rigidity) and the resistance to the actin flow is maximum. In both slip and catch cases, the optimal rigidity obtained in the clutch model (Fig. A1) is in agreement with previous experimental and theoretical results [1, 2]. Below the optimal rigidity, are the load-and-fail cycle rules. As the stiffness of the substrate increases, the average force at the bound binders increases (see below) and so does the traction.

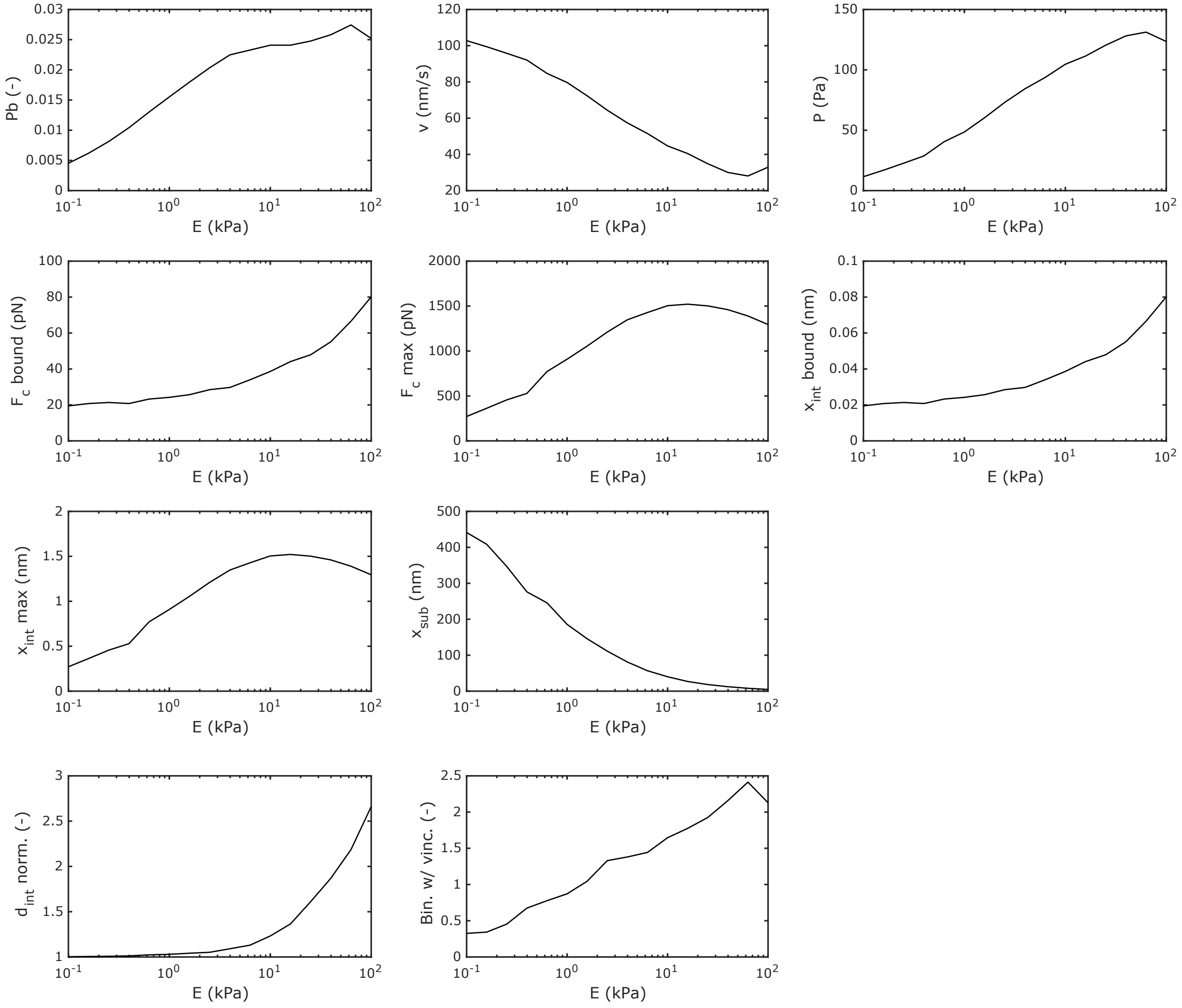

Fig A2. Effect of reinforcement by talin unfolding in cell adhesion.
For the model with talin reinforcement, plot against Young's modulus of the substrate E of the variables P_b, v, P, F_c over bound binders, F_c^{max}, x_{int} over bound binders, x_{int}^{max}, x_{sub}, $d_{int,norm}$ and # binders with vinculin. All variables increase monotonically as the substrate stiffness increases, including the cell tractions, in agreement with previous data, except for the actin velocity v and the substrate displacement x_{sub} instead decrease. As in previous cases at low substrate stiffnesses, the forces at each molecular chain are low, talin mechanosensing does not activate and, as a result, integrin recruitment does not occur. Therefore, results are comparable to the catch case above A1. In stiff substrates, above, ≈ 10 kPa, we see an increase in all the variables that decreased in the catch case, including the cell tractions. As the substrate stiffness increases, the forces at each molecular chain increase, which activates talin unfolding. If talin exposes its VBS, vinculin binds to the talin rod and fosters integrins recruitment within the AC, i.e. the integrin density d_{int} increases, and so does the binding rate, which eventually increases the probability of bound binders and the cell traction P. This process explains the adhesion reinforcement by talin mechanosensing [2, 6].

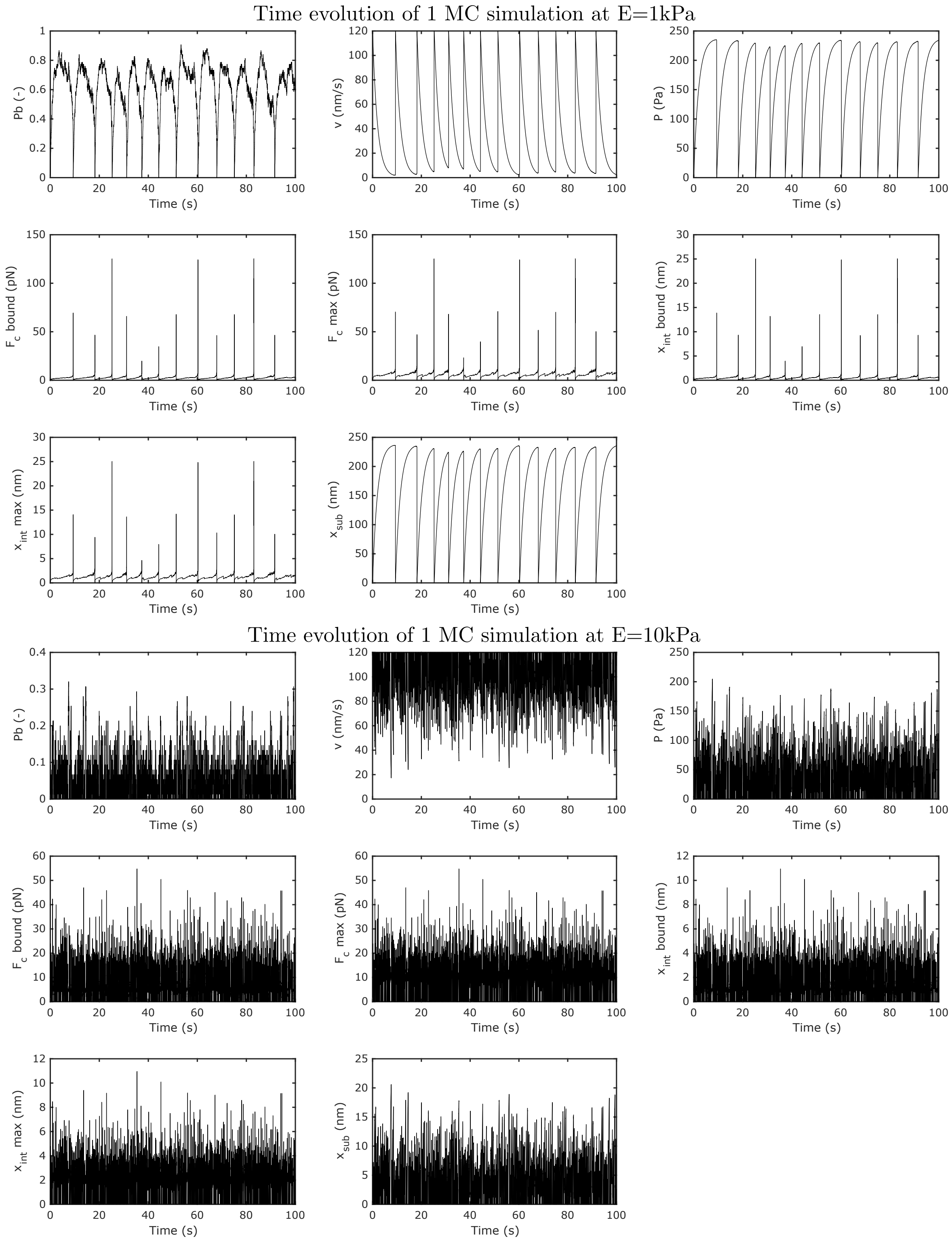

Fig A3. Time evolution of the slip case.

Time evolution of the slip case for the variables P_b, v, P, F_c over bound binders, F_c^{max}, x_{int} over bound binders, x_{int}^{max} and x_{sub}, for $E = 1\,\text{kPa}$ and $E = 10\,\text{kPa}$. Model parameters are given in Table A1. The lifetime of the adhesion clusters is considerably shorter for stiffer substrates.

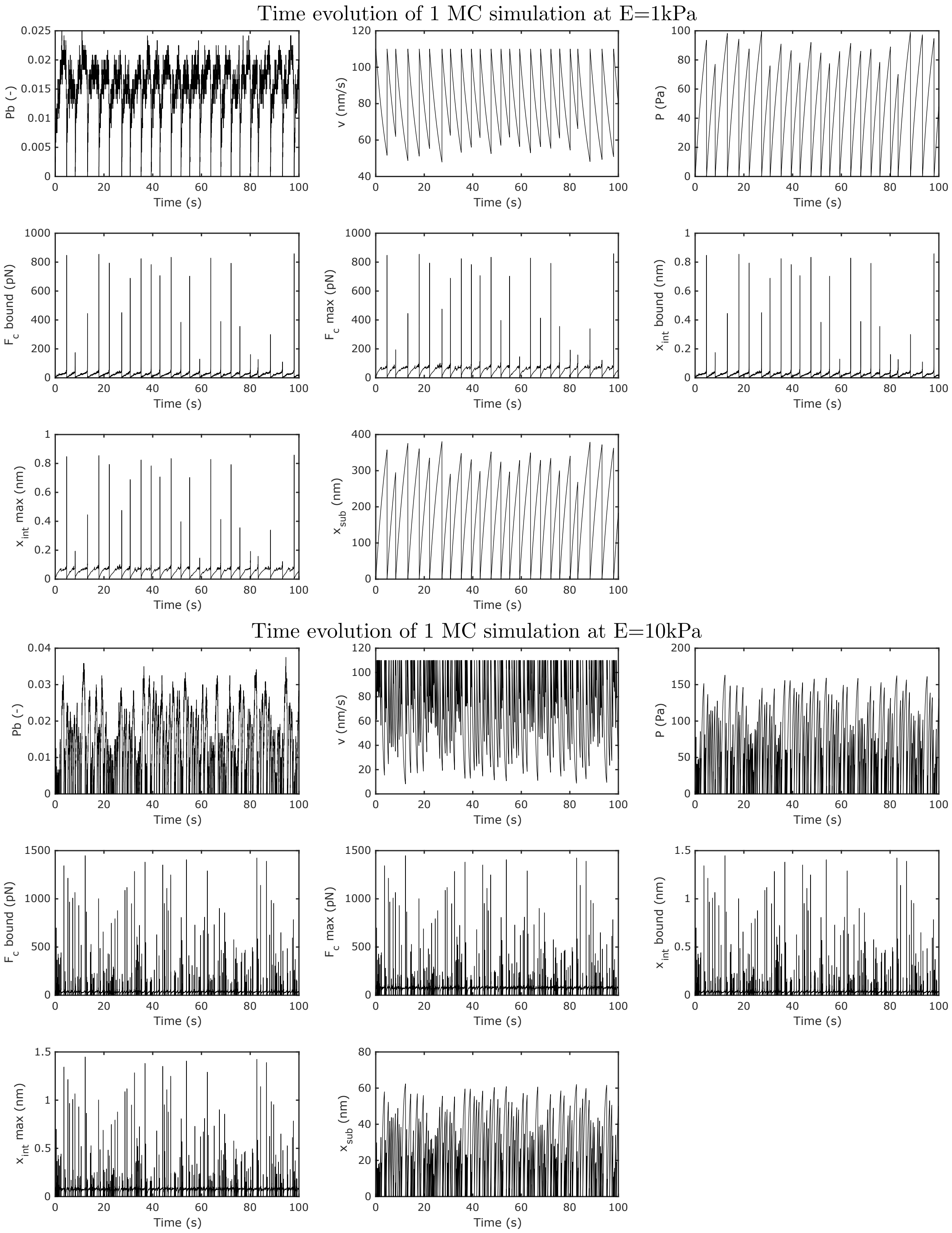

Fig A4. Time evolution of the catch case.
Time evolution of the catch case for the variables P_b, v, P, F_c over bound binders, F_c^{max}, x_{int} over bound binders, x_{int}^{max} and x_{sub}, for $E = 1\,\mathrm{kPa}$ and $E = 10\,\mathrm{kPa}$. Parameters from Table A1. The MC loops to get a cluster completely made of free binders are considerably shorter for stiffer substrates.

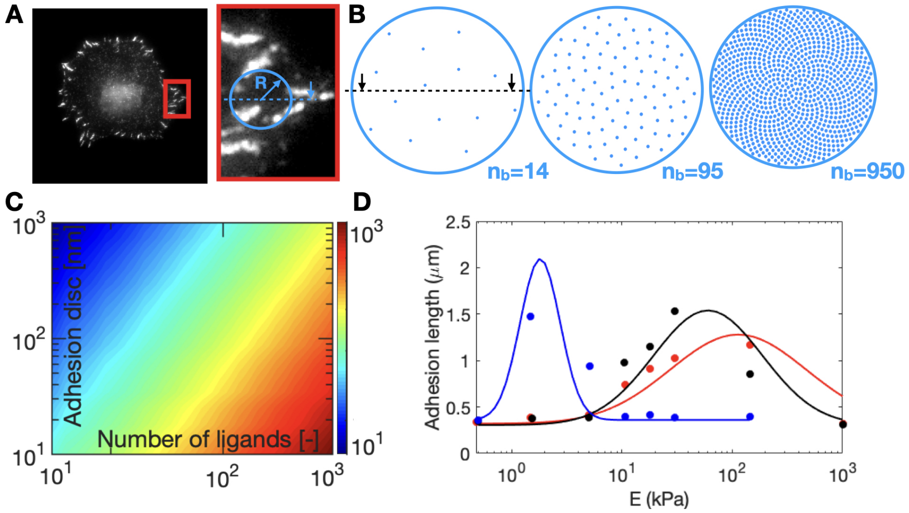

Fig A5. Number of equispaced ligands n_c as a function of the adhesion radius.
We compute a set of n distributions for n_c=1 to n_c=5000 for a radius of μm. Then we extend the set by scaling each one of the distributions by a different number of radii, m, from 10nm to 1μm. Altogether, we obtain a set of nxm distributions of n_c ligands within adhesion of varying radii. (a) Cell expressing AC. Inset of the front cell. (b) Different number of ligands in an AC of the same area. (c) Computation of the ligand's distance (color map) as a function of the adhesion size and number of ligands. Among all the combinations of several ligands and radii, we obtain sets of ligand distances that are not physically viable. Integrins have an approximate equivalent radius of 2.5nm, based on an estimated footprint of the integrin dimer [22]. Considering a dense packing of the integrins, the maximum density is ≈ 25,000 integrins/μm^2. Therefore, integrins can not be apart less than ≈ 5nm, which we impose as a physical constraint in the spacing between ligands. Furthermore, we also constrain the spacing by the number of actin filaments that fit in a given domain of radius R. Assuming a stress fiber of up to 30 actin filaments, an average filament diameter of 20 nm, and a distribution almost parallel to the contact plane, there is a limit of bound ligands distance of 20 nm. In ventral-like adhesions, the angle of actin and talin with respect to the membrane has been measured in 2-6 and 15°, respectively [19]. To accommodate the actin filaments within the stress fiber and assuming a stoichiometry of talin-integrin of 1:1 and talin to actin of 1:1 to 1:3 (three talin domains can link to actin filaments) we get a range of possible ligand distances that goes up to ≈ 400 nm. (d) Experimental data [21] and fitting of the adhesion size for different ligands spacing as a function of the substrate stiffness.

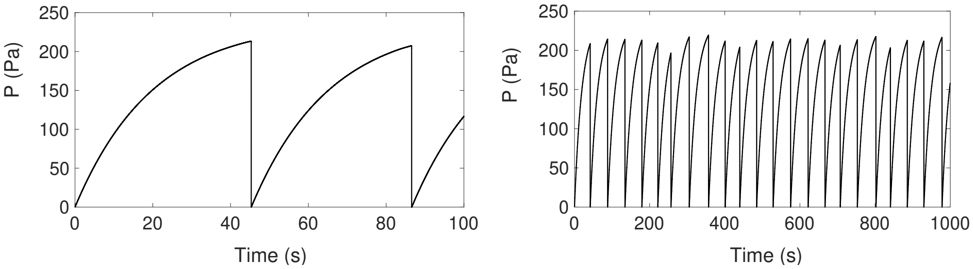

Fig A6. Comparison of the number of MC simulations for two different final times $t_f = 100\,\mathrm{s}$ and $t_f = 1000\,\mathrm{s}$.
We run a slip case and fix Young's modulus of the substrate $E = 0.1\,\mathrm{kPa}$. As the MC simulations are concatenated, to do the average over MC, we do the average in time of the variable. For $t_f = 1000\,\mathrm{s}$, the average of the cell traction is $P \approx 138\,\mathrm{Pa}$, while for $t_f = 100\,\mathrm{s}$ the average is $P \approx 132\,\mathrm{Pa}$.

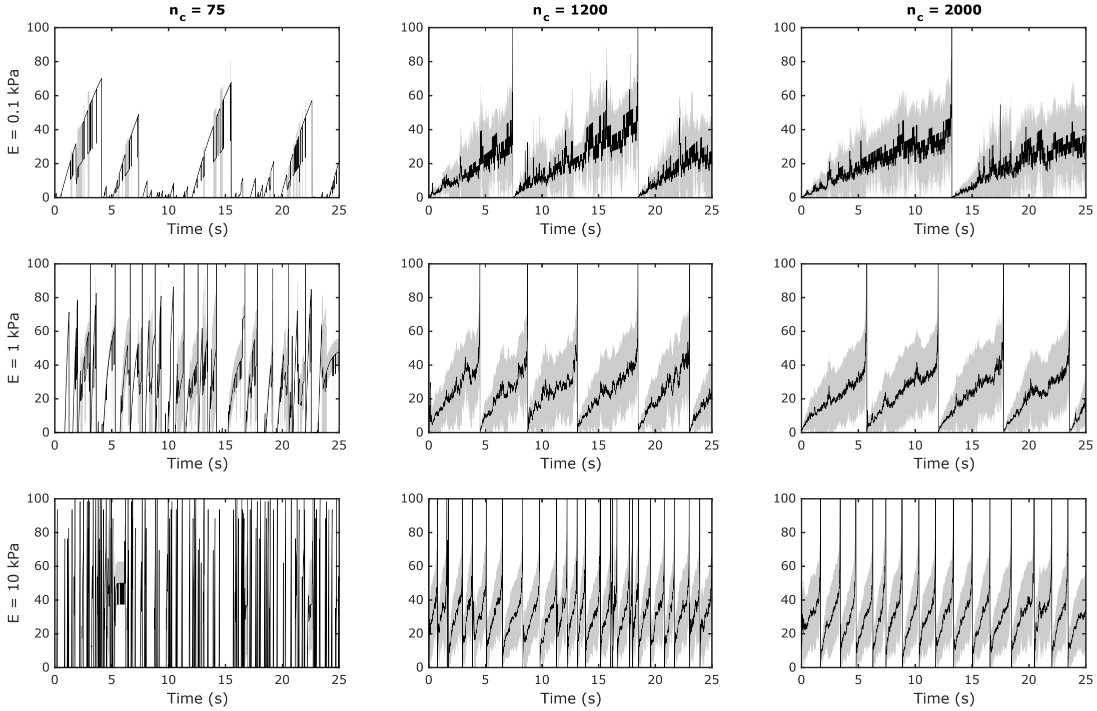

Fig A7. Mean and standard deviation of the force F_c (pN) computed over bound binders.
Three values of Young's modulus (E =0.1, 1 and 10 kPa, in rows) and three values of a number of binders $n_c = n_m$ ($n_c = 75$, $n_c = 1200$, $n_c = 2000$, in columns). The rest of the parameters are as the default values in Table A1. Standard deviation in light grey. For visualization purposes, the final time is fixed to $t_f = 25$ s and the y-limit for F_c is set to 100 pN. As the substrate stiffness increases, the average force F_c slightly increases for all three values of n_c. As we increase the substrate stiffness, we see an increase in the number of cycles for a fixed $t_f = 25$ s. The increase in the averaged force is mostly due to force peaks that appear at the end of each cycle. If the number of cycles increases, as it happens when the stiffness of the substrate increases, the average force increases. Therefore, the peaks obtained at the end of each cycle hide and distort the actual force at the molecular binders.

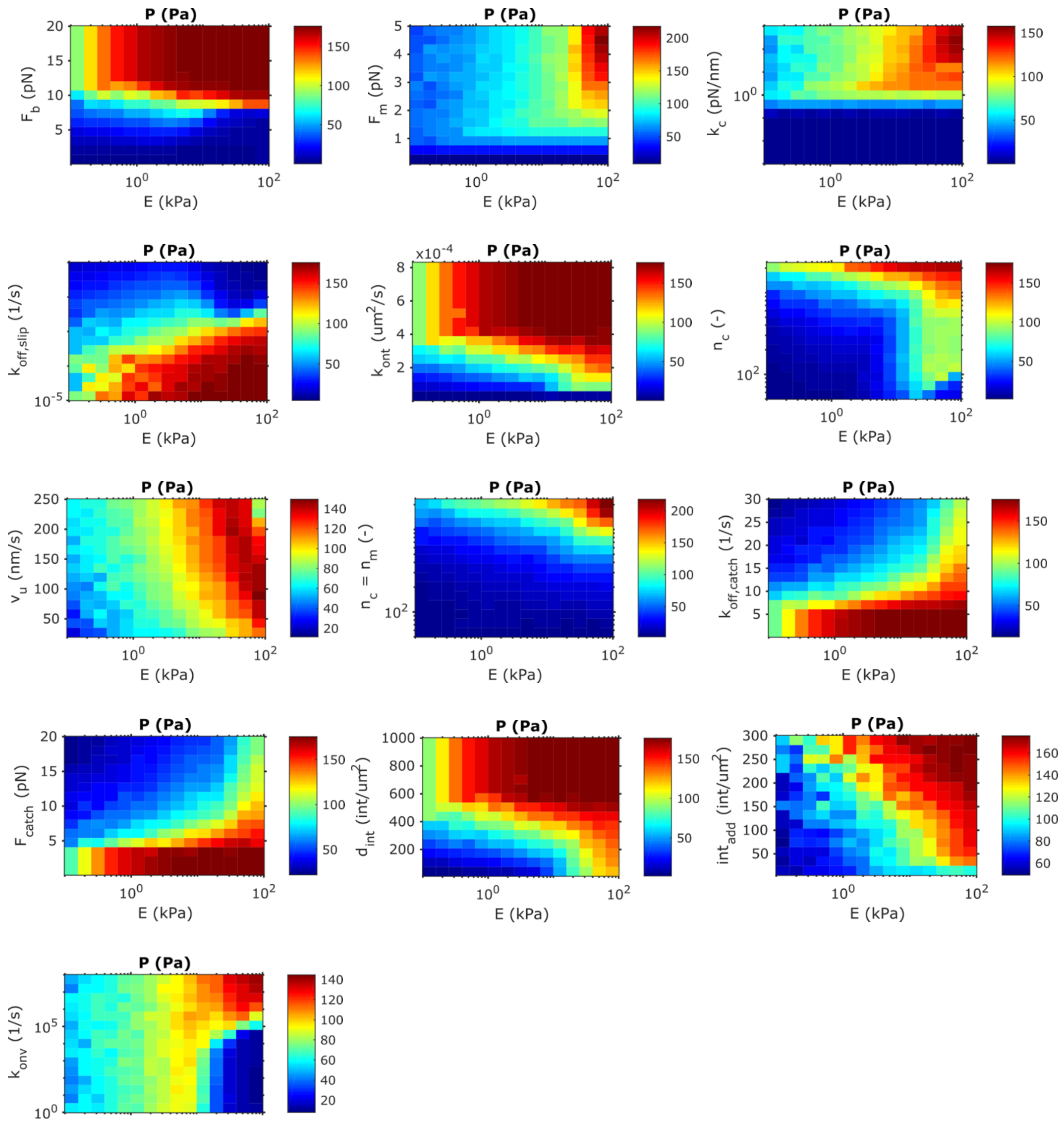

Fig A8. Sensitivity analysis of the reinforced case.

For the model with talin reinforcement, values of the cell traction P following colormap, against Young's modulus E, varying in a suitable range the parameters F_b, F_m, κ_c, $k_{off,slip}$, k_{ont}, n_c, v_u, n_c kept equal to n_m, $k_{off,catch}$, F_{catch}, d^0_{int}, int_{add}, k_{onv}. The results show a response to the model parameters similar to the non-reinforced model for low stiffnesses of the substrate, where the talin reinforcement has not taken place due to the low force in each clutch (see the main text). For the upper part of the ranges and all model parameters, the cell traction P always increases as Young's modulus of the substrate increases. This change with respect to the drop in traction forces for the catch case is due to the integrin recruitment when vinculin binds to the unfolded talin. In other words, the system never gets into the frictional slippage regime. In terms of the parameters that control the integrin recruitment, we see that increasing int_{add} the cell traction P corresponding to the optimal stiffness increases. This is because the integrin density added at each recruitment step increases the total integrin density and, consequently, the on-rate of integrin binding increases. Similarly, an increase in the on-rate of vinculin to talin, k_{onv}, results in an increase in the cell traction P in the upper part of the stiffness range, while at lower stiffnesses there is no effect. This is because integrin recruitment is only activated once talin is unfolded, which only occurs at high stiffnesses. We also see that integrin recruitment is only effective for values of $k_{onv} > 10^5$. Below this value, integrin recruitment does not happen and we observe a decrease in cell traction similar to the catch case without reinforcement (see main text).
