# Supplementary material for: A multi-scale clutch model for adhesion complex mechanics
Source: PLoS Comput Biol. 2023 Jul 14;19(7):e1011250. doi: 10.1371/journal.pcbi.1011250 (PMC10393167; doi:10.1371/journal.pcbi.1011250)
Supplement: Attachment — Submitted filename: R1_Venturini.pdf [file pcbi.1011250.s003.pdf]

Reviewer #1

In this paper, Venturini et al propose a new computational model in order to evaluate the contribution of single adhesion proteins to the clutch behavior. This is an important and unanswered question in the field of Integrin-based adhesions. The proposed computational model is based on on a mechanical system comprising elements mimicking integrin, talin, actin and a substrate of variable stiffness. These elements interact with one another governed by kinetic rates and transmit stresses and deformations which, in turn, strengthen or weaken the relative interactions.

We would like to thank the reviewer for the revision of our work. We think there are important points raised by the reviewer that have made our manuscript improve and more understandable.

Topic and question

-While the main idea behind this work is important and the exact interplay of several adhesion proteins to the clutch behavior is currently unknown, the study does not seem to pose and answer a clear scientific question and/or hypothesis regarding the complexity of mechanisms involved.

Thank you for raising this concern. Before describing the main scientific question, we would like to note that this contribution is not only about the scientific questions, which of course are essential but also about the development of a new model, based on the classical clutch model, that can be used to computationally address many future scientific questions on cell adhesion. Indeed, the reviewer acknowledges the importance of how exactly the interplay of several adhesion proteins is and that it is currently unknown. Our model aims at providing a computational tool capable of addressing this issue. We believe that this is, by itself, a key contribution to this work. To this end, we will make our code available for reuse by other researchers. The link to the repository is now available in the updated version of the manuscript.

The main scientific question that we focused on in this work is how forces are transmitted in the adhesion complex along the different adhesion molecules that form the adhesion complex. Specifically, we focus on integrins and talin molecules, actin filaments, and the ECM, and, for these molecules, we analyze kinetic rates of binding and unbinding, conformational changes, exposure of actin-binding sites in the talin rod, and force transmission. Although detailed data are available in the literature for those individual molecules alone, such a precise measurement within the adhesion complex is currently unknown, as the reviewer pointed out above.

Although that is the main scientific question we wanted to address with our model, we also used the model to understand how the ECM deforms locally, and how different types of integrins molecules change the adhesion complex dynamics under ECM rigidities and in time.

We have modified the section describing the goals of our work (page 6) to clearly describe our goals.

-The authors state: “We hypothesize that an improvement in the molecular characterization within the clutch models could answer these questions” . However, the five questions that the model wants to address are somehow vague, therefore it’s hard to understand the significance of the results. As an example, the question “How do individual molecular chains within the AC behave in different substrate stiffnesses?” does not refer to any specific protein (talin, integrin, or vinculin)

and does not specify a specific type of behavior (kinetic rates, conformational change, exposure of binding sites, force transmission, unfolding). Therefore, once all proteins are put together into one model, it still remains unclear to me what is the exact question regarding their interplay.

We agree the questions were somehow vague. However, we would like to note that we were not specifically addressing all those questions. That is why we phrased the sentence with a "For example…" at the beginning, just to pose general questions, that include all aspects specified by the reviewer, that are still not clear in the field. We have now rewritten that section of our article (page 6), to clearly describe what we focus on, and how we do it, as we also discussed in the previous comment.

Referring to the question "How do individual molecular chains within the AC behave in different substrate stiffnesses?", this is actually the main question that we aimed to address. The behavior of these molecules alone has been studied in detail but what are the mechanical, conformational, and binding states in an adhesion complex are still not well known. We have now specifically referred to the main adhesion molecules, which include integrins and talin, actin filaments, and the ECM. Note that there is an important improvement here in comparison with previous clutch models because we have introduced a very detailed mechanical model of the talin molecule within the adhesion model. In terms of the specific behaviors that the reviewer mentions, we are also interested in all those behaviors and we have included them in our model (kinetic rates of binding and unbinding, conformational change in the integrin and talin molecules, exposure of actin-binding sites, and force transmission across these molecules), which we have now explicitly mention in the current version of the manuscript.

Second, how this model improvement is intended is not explained clearly. The method is presented at the end of the paper and some details are missing. For example, the authors state that they use Monte Carlo or Gillespie simulations. Which results are produced with one method and which results are produced with the other method? Also, why they use two methods? I invite the authors to focus the scope of the simulations and reorganize the results in order to answer a more specific scientific question, that can be directly discussed in the context of previous knowledge. I also invite the authors to explain their methods in more details and present it before the results.

We introduced the model at the end of the article because this is the location where methods or models (we have changed now the title to "Models") are located in PloS Computational Biology:

https://journals.plos.org/ploscompbiol/s/submission-guidelines#loc-layout-and-spacing

Actually, we agree that in some contributions, including ours, would be perhaps more reasonable to add this section before the results. We have asked PloS Computational Biology about the possibility of including the models before the results and we have now the consent to do it, which is now done in the current version of the manuscript.

Regarding the use of Monte Carlo and Gillespie simulations, we used the Monte Carlo method in the simulations in all the results related to the previous clutch models (section Models for cell adhesion and goal of this work and Results of the clutch model in the SI). There was an error in the description in the Methods section. It has been corrected now. The Gillespie algorithm is used in all other simulations of our multi-scale model. We used the Monte Carlo simulation because it was the method used in previous works so it was more straightforward for us to compare with previous computational results. Then, we change to the Gillespie algorithm in the multi-scale

clutch model because the previous model of the talin rod (Yao et al.) used this approach and it was now more straightforward for us to use this method so that we can compare in detail the conformational changes in the talin rod with those results shown by Yao and co-workers.

We have reviewed the Models section again and reorganized the section. We think that now the description is self-consistent and all the details of the model are described.

Further questions about the explanation of the methods:

-at the end of pang. 17, the authors state: dt is the time step in the Monte Carlo or Gillespie simulation(see Section). What section?

Sorry for this typo. We meant the section below. We have rewritten this sentence.

-Regarding how the elements in the model are treated, are the engaged versus disengaged clutches considered separately in the simulations? is the force distributed only on the engaged clutches or on all clutches?

Yes, the engaged and disengaged clutches are considered separately in the simulations because force can only be transmitted along the engaged clutches. The molecular chains that are not connected to the ECM should not be able to transmit forces to it. This is the reason why the sum in Eq. (3) performed only over n_{eng}, the number of engaged clutches.

Results

-It's hard to follow what is the main message of each figure. For example, in Fig. 8 several threshold values for combinations of substrate rigidity, force and and rate constants are identified. Are they meaningful? can these be related to any experimental observations or previous models?

Each figure is associated with one section of the manuscript. The rationale behind each section and figures are as follows:

1. In the first subsection, "Role of the ECM rigidity on a single adhesion chain" (and Fig. 2) we built the model of a single molecular chain because it will allow us to later build the full model. It also provides a simpler picture of one single adhesion chain, as we describe in the text. The results of this section are in agreement with those shown by Yao et al. (24), can be compared with results at the single talin rod scale, and show how a talin molecule would behave in a simplified model where myosin motors pull on an actin filament attached to the talin rod.
2. In the next section (and Fig. 4-5), we extended the model to consider one adhesion complex made of many adhesion chains. This is related to full adhesion complex, e.g., focal adhesions. First, we focus on adhesion complexes crowded by two specific integrins in Section "Role of the ECM rigidity in α5β1 and αV β3-crowded focal adhesions". These are well-known integrin types. Here, we showed how forces are transmitted across each adhesion molecule, and how the conformational changes in integrins and talin are for substrates of different stiffnesses (section "Adhesion dynamics in α5β1 and αV β3-based adhesion complexes"). We believe this is a key result of our model and, as far as we know, no other theoretical or experimental work has shown the force, binding and conformational state of these molecules within the adhesion complex in such detail.
3. In the next section, "Integrins behavior in AC dynamics" (and Fig. 6) we vary the main model parameters of the integrins to understand how the adhesion complex would behave

when crowded by integrins of different types. This is an important study because different cell types express one or another type of integrins. Future experimental data on single integrin molecules and adhesion complexes should be used to verify these results.

4. Finally, because our model can also take into account the distance between ligands to model ECM deformation point-wise, thanks to the use of Green's functions, we use the model to reproduce experimental data.

These four points are directly aligned with the goal of our article: understand and quantify how kinetic rates of binding and unbinding, conformational changes, exposure of actin-binding sites in the talin rod, and force transmission of integrins and talin molecules behave within an adhesion complex. We don't see any confusion with this history line and we think it is the right way of building our results. However, to make clear what are the messages of each section and figure, we have added a new paragraph before the results section in the current version of the manuscript.

Regarding the specific question about Fig. 8, yes, we believe these values are meaningful. In the previous sections, we discussed the specific adhesion behavior when two specific integrins, α5β1, and αVβ3, were considered. However, there are many other integrin types involved in cell adhesion which most probably have similar mechanical and kinetic properties, but still with certain variations. However, there are no experimental results that can be used to describe the model parameters. This is actually why we ran a sensitivity analysis for the model parameters that describe the integrin behavior. This figure and section aim to understand how cell adhesion would change when the integrin behavior changes.

-On page 16, the authors state: "our results on the deformation and force in each CAM closely also agree with previous data". Here a reference is needed. The reader doesn't know what data is this sentence referring to.

Sorry about not being specific about this. We explain these aspects in the section "Force distribution and architecture in integrin-based cell adhesions". We didn't want to repeat again all the discussion on these data because we should discuss again the force and displacement of the talin and the integrin molecules, which is almost 2 paragraphs. Following the reviewer's suggestion, we have now referred to that subsection in the Introduction.

-Some predictions of the model have not been and cannot be experimentally tested. This limitation put into question the validity of the model itself. I invite the authors to more clearly list what results are validated and what results are predictions for future studies. For the predictions, I suggest making a case for why these results should be true based on indirect evidences in the field.

We are not aware that any of the results we presented could not be tested experimentally. What specific data is the reviewer referring to? Perhaps there are experiments difficult to perform, or even not doable today. Honestly, we don't think this is an issue. But we cannot think of any limitation why these are not doable now or in the near future. Even so, we don't understand why the reviewer thinks that it would question the validity of the model. Thousands of theoretical results have paved the way for further experimental results in the past, which we believe is a nice communication between theoretical and experimental work. We understand that validity refers to whether the model is well-founded and likely corresponds accurately to the real world. We have based our model on well-established models at different scales, which are in agreement with experimental data, and we think that establishes the validity of our model. Another issue is whether we can verify the model. In this regard, we think there are results that verify our model: 1) our model reproduces the traction force exerted by the cell, similar to the classical clutch model (see

Fig. 4). 2) our model reproduces the force values measured at the integrin and talin levels (see discussion at the introduction and Fig. 11). This is an important result not only because improves previous clutch model predictions but because indicates that the model provides realistic values. From our point of view, this is the evidence in the field that indicates that our results are realistic. However, what specific force at each molecule is within the adhesion complex is a model prediction that will need to be verified by experimental data in the future.

We have now included a paragraph about these issues in the Conclusions section of the current version of the manuscript.

Writing and presentation

The manuscript needs language editing. The SI is missing several equation numbers through the text.

We apologize for any language issues. We have carefully reviewed the entire document, check through two grammar checker software, and made several changes. We hope that the article writing is now acceptable.

Reviewer #2

The manuscript "A multi-scale clutch model for adhesion complex mechanics" by C. Venturini and P. Sáez develop a multi-scale computational for adhesion complexes based on the classical approaches of the clutch model. The model includes the typical parameters of the clutch models such as retrograde flow, myosin motors but two new ingredients (integrins and talin) are deeply analyzed. The multi-scale model dissects the role of integrins and talin at the molecular level, providing information about forces are transmitted through single chains. On top of the common proxies of the molecular clutch model as tractions and retrograde flow, the model shed light on the forces and elongation for integrins and talin. Authors start meticulously by studying the role of talin in the absence of integrins. After, they show how different integrins behave and how parameters that are unique to each type of integrins such as binding/unbindings rate can impact the force transmission in single chains and ultimately determine rigidity sensing. Finally, they use the model to study how the ligand spacing impacts force transmission and adhesion formation. The manuscript is well-written, the data is carefully presented, and the model results are validated and in good agreement with previous experimental results. The parameters of the model are well explained, and the assumptions are supported by the current literature.

Thank you for your summary and assessment of our work.

Please, note that following the journal guidelines the link to the repository of our code is now available in the updated version of the manuscript.

Considering my previous comments, I do suggest the acceptance of the manuscript. While this reviewer understands the simplicity of the model, I would like to raise two points that it would be interesting if the authors can discuss.

1. Talin presents four binding sites for integrins in its dimeric form.

2. Different beta integrin tails may show diverse affinities for talin binding (*Mol. BioSyst.,* 2014, 10, 3217)

Can the authors speculate how the model results are affected by that? Can these variables be introduced in the model?

These are very interesting questions. Regarding the integrin binding sites to talin, we were actually not aware of these 4 binding sites. We knew about two possible binding sites, as discussed by Calderwood (see ref 27 in the main text), one in the head and one in the rod. We included the binding of integrins with the head of the talin molecule because it was not clear to us how the other binding site affects mechanically the adhesion complex. Perhaps the reviewer can share any publication showing these other binding sites because we were not able to find information about it. In any case, it would be relatively simple to introduce mechanical effects in the adhesion complex. However, we should have either a clear idea of what the mechanical effect is or, at least, a clear idea of the effect in the mechanical behavior of the complex so that we can use the model to propose or rule out mechanical interactions between molecules. Unfortunately, we don't have any of these two indications.

Regarding the diverse affinities for talin binding to the different beta integrin tails, we assume the binding rate of integrins to the ECM is faster than other binding rates. And not only the binding

rates would be relevant here, but also the unbinding rates. In this case, the integrin-talin link is usually considered the weaker link, and we assume the other links are more stable than this one. Again, this is a modification that should be difficult to introduce and analyze in the future.

We cannot predict the results, and we would prefer not to speculate about it, but we have added a few sentences in the discussion of the current version of the manuscript discussing these two aspects. If the reviewer can share an article about those 4 binding sites, we will be happy to add it to our discussion.
