# Supplementary material for: A multi-scale clutch model for adhesion complex mechanics
Source: PLoS Comput Biol. 2023 Jul 14;19(7):e1011250. doi: 10.1371/journal.pcbi.1011250 (PMC10393167; doi:10.1371/journal.pcbi.1011250)
Supplement: Attachment — Submitted filename: Review_Saez.docx [file pcbi.1011250.s002.docx]

The manuscript “A multi-scale clutch model for adhesion complex mechanics” by C. Venturini and P. Sáez develop a m­­ulti-scale computational for adhesion complexes based on the classical approaches of the clutch model. The model includes the typical parameters of the clutch models such as retrograde flow, myosin motors but two new ingredients (integrins and talin) are deeply analyzed. The multi-scale model dissects the role of integrins and talin at the molecular level, providing information about forces are transmitted through single chains. On top of the common proxies of the molecular clutch model as tractions and retrograde flow, the model shed light on the forces and elongation for integrins and talin. Authors start meticulously by studying the role of talin in the absence of integrins. After, they show how different integrins behave and how parameters that are unique to each type of integrins such as binding/unbindings rate can impact the force transmission in single chains and ultimately determine rigidity sensing. Finally, they use the model to study how the ligand spacing impacts force transmission and adhesion formation. The manuscript is well-written, the data is carefully presented, and the model results are validated and in good agreement with previous experimental results. The parameters of the model are well explained, and the assumptions are supported by the current literature.

Considering my previous comments, I do suggest the acceptance of the manuscript. While this reviewer understands the simplicity of the model, I would like to raise two points that it would be interesting if the authors can discuss.

1. Talin presents four binding sites for integrins in its dimeric form.

2. Different beta integrin tails may show diverse affinities for talin binding (*Mol. BioSyst.,* 2014, 10, 3217)

Can the authors speculate how the model results are affected by that? Can these variables be introduced in the model?
